# SpoT-Mediated Regulation and Amino Acid Prototrophy Are Essential for Pyocyanin Production During Parasitic Growth of *Pseudomonas aeruginosa* in a Co-culture Model System With *Aeromonas hydrophila*

**DOI:** 10.3389/fmicb.2018.00761

**Published:** 2018-04-18

**Authors:** Nina Jagmann, Bodo Philipp

**Affiliations:** Institute for Molecular Microbiology and Biotechnology, University of Münster, Münster, Germany

**Keywords:** *Pseudomonas aeruginosa*, *Aeromonas hydrophila*, pyocyanin, quorum sensing, stringent response, amino acids, co-culture

## Abstract

The opportunistic pathogen *Pseudomonas aeruginosa* employs its complex quorum sensing (QS) network to regulate the expression of virulence factors such as pyocyanin. Besides cell density, QS in this bacterium is co-regulated by environmental cues. In this study, we employed a previously established co-culture model system to identify metabolic influences that are involved in the regulation of pyocyanin production in *P. aeruginosa*. In this co-culture consisting of *P. aeruginosa* and the chitinolytic bacterium *Aeromonas hydrophila*, parasitic growth of *P. aeruginosa* is strictly dependent on the production of pyocyanin. We could show that in this co-culture, pyocyanin production is likely induced by the stringent response mediated by SpoT in response to nutrient limitation. Pyocyanin production by stringent response mutants in the co-culture could not be complemented by overexpression of PqsE. Via transposon mutagenesis, several amino acid auxotrophic mutants were identified that were also unable to produce pyocyanin when PqsE was overexpressed or when complementing amino acids were present. The inability to produce pyocyanin even though PqsE was overexpressed was likely a general effect of amino acid auxotrophy. These results show the value of the co-culture approach to identify both extra- and intracellular metabolic influences on QS that might be important in infection processes as well.

## Introduction

The Gram-negative Gammaproteobacterium *Pseudomonas aeruginosa* exhibits a broad metabolic versatility (Clarke, 1982; Alonso et al., 1999) and exists in a wide variety of environmental habitats (Ringen and Drake, 1952; Hardalo and Edberg, 1997; Mena and Gerba, 2009). As opportunistic pathogen, *P. aeruginosa* is able to infect different host organisms (Rahme et al., 1995; Hilbi et al., 2007; Morales et al., 2011), and it is a major agent of human infections posing a particular threat to immunocompromised persons and patients with cystic fibrosis (Driscoll et al., 2007; Harrison, 2007). Within a complex regulatory network that allows *P. aeruginosa* to sense and respond to different environmental cues with changes in gene expression, quorum sensing (QS) is a key regulatory system of this bacterium rendering *P. aeruginosa* a model organism for QS (Williams and Camara, 2009; Coggan and Wolfgang, 2012; Jimenez et al., 2012).

Quorum sensing (QS), a process of cell-to-cell communication, describes the regulation of gene expression in response to signal molecules, which are mostly membrane-diffusible *N*-acyl-homoserine lactones (AHLs) in Gram-negative bacteria. Usually, QS is defined to be cell density-dependent, and an increase in cell density is accompanied by an accumulation of signal molecules to a certain threshold concentration, at which the signals bind to the cognate receptor proteins, thereby eliciting changes in gene expression (Williams, 2007). In *P. aeruginosa*, however, QS is to a large degree co-regulated by environmental signals, mostly nutritional cues, resulting in the differential regulation of QS-controlled genes under different nutrient conditions (van Delden et al., 2001; Wagner et al., 2003; Bazire et al., 2005; Duan and Surette, 2007; Palmer et al., 2007; Oglesby et al., 2008). In addition to extracellular metabolic information, it is hypothesized that intracellular metabolic influences may be involved in QS regulation as well and that changing ratios of macronutrients may be monitored via intracellular metabolite fluxes (Mellbye and Schuster, 2014).

In this study, we aimed at investigating such extra- and intracellular metabolic influences that are involved in QS regulation in *P. aeruginosa*. For this, we employed a co-culture model system consisting of *P. aeruginosa* and the chitinolytic bacterium *Aeromonas hydrophila*. Chitin, which cannot be utilized by *P. aeruginosa*, is the sole growth substrate in this co-culture, and *P. aeruginosa* strictly depends on the production of virulence factors in order to grow by parasitically exploiting the chitinolytic properties of *A. hydrophila* (Jagmann et al., 2010, 2016). This is mediated by the redox-active virulence factor pyocyanin, which blocks the citric acid cycle in *A. hydrophila* by inhibiting the enzyme aconitase through the formation of reactive oxygen species, leading to a massive release of acetate by this bacterium. Acetate then serves as growth substrate for *P. aeruginosa*, while cells of *A. hydrophila* become inactivated. In a previous study employing the co-culture we identified the guanidinobutyrase GbuA that may take part in an intracellular metabolite flux involved in QS regulation in *P. aeruginosa* (Jagmann et al., 2016).

*Pseudomonas aeruginosa* possesses three QS system, which are closely interrelated and coordinate the expression of virulence factors. The Las and Rhl systems are mediated by AHLs as signal molecules and comprise the respective signal synthases (LasI and RhlI) and receptors (LasR and RhlR) (Williams, 2007). The PQS system is mediated by the 2-alkyl-4(1H)-quinolones (AQs) 2-heptyl-4-quinolone (HHQ) and 2-heptyl-3-hydroxy-4-quinolone (Pseudomonas Quinolone Signal: PQS) (Dubern and Diggle, 2008). HHQ is synthesized by the gene products of *pqsABCDE* and is further converted to PQS by the monooxygenase PqsH (Gallagher et al., 2002; Deziel et al., 2004; Drees and Fetzner, 2015). The *pqsABCDE* operon is regulated by the transcriptional regulator PqsR, which binds both HHQ and PQS (Xiao et al., 2006). PqsE mediates the cellular response to AQ signaling in a yet unknown way and is, thus, required for the production of virulence factors, such as pyocyanin (Rampioni et al., 2007). In the co-culture with *A. hydrophila*, the Rhl and PQS systems are required for pyocyanin production by *P. aeruginosa* (Jagmann et al., 2016), and it has been demonstrated that various mechanisms integrate nutrient limitation into QS activation mainly at the level of these two systems (Mellbye and Schuster, 2014; Welsh and Blackwell, 2016).

Phosphate limitation is integrated via the two component system PhoR-PhoB leading to an induction of both the Rhl and PQS systems (Jensen et al., 2006), which is mediated via a fourth QS signal that was termed IQS [“integrating QS and stress response”; (Lee et al., 2013)]. Transcription of both systems is enhanced under iron limitation as well (Jensen et al., 2006; Schmidberger et al., 2014).

Carbon limitation and the resulting shortage of amino acids is integrated via the stringent response, which is a conserved bacterial stress response enabling bacteria to adapt to growth-inhibiting environmental stresses (Magnusson et al., 2005; Potrykus and Cashel, 2008). It is mediated by the guanosine nucleotides pppGpp and ppGpp as regulatory components. Accumulation of (p)ppGpp is regulated by two enzymes, the monofunctional synthetase RelA and the bifunctional synthetase/hydrolase SpoT. In P. aeruginosa, (p)ppGpp is required for full expression of the Rhl and Las systems and negatively regulates the PQS system (van Delden et al., 2001; Schafhauser et al., 2014).

Nitrogen limitation leads to an induction of rhamnolipid biosynthesis via the small RNA NrsZ together with the two-component system NtrB/C and the alternative sigma factor RpoN without involvement of the Rhl system as it was previously suggested (Medina et al., 2003; Wenner et al., 2014). However, upstream regions of both *rhlR* and *rhlI* harbor RpoN-binding sites (Medina et al., 2003; Thompson et al., 2003), and RpoN has been shown to regulate PqsR (Cai et al., 2015), making a connection between nitrogen limitation and Rhl and PQS system induction possible as well.

To investigate metabolic influences that are involved in QS regulation, we used the production of pyocyanin by *P. aeruginosa* in the co-culture as an indicator for Rhl and PQS system activity. First, we wanted to elucidate which environmental cues induce QS in *P. aeruginosa* in the co-culture, and, second, we wanted to identify potential intracellular metabolic influences that are involved in the regulation of QS.

## Materials and Methods

### Bacterial Strains, Plasmids, and Growth Experiments

Bacterial strains and plasmids used in this study are shown in Supplementary Table S1 (Supporting Information). *P. aeruginosa, A. hydrophila*, and *Escherichia coli* strains were maintained on solid [1.5% (w/v) agar] lysogeny broth (LB) plates. Antibiotics were used at the following concentrations: ampicillin (100 μg ml^-1^ for *E. coli*), carbenicillin (400 μg ml^-1^ for *P. aeruginosa*), tetracycline (10 μg ml^-1^ for *E. coli*; 160 μg ml^-1^ for *P. aeruginosa*), chloramphenicol (30 μg ml^-1^ for *E. coli*; 350 μg ml^-1^ for *P. aeruginosa*), and gentamicin (20 μg ml^-1^ for *E. coli* and 120 μg ml^-1^ for *P. aeruginosa*).

Cells were cultivated in a volume of 4 ml in 15 ml glass tubes at 200 r.p.m. in a rotary shaker. For experiments with auxotrophic mutants, co-cultures with these mutants and the wild-type were incubated at 30°C, whereas co-cultures with all other strains were incubated at 37°C. Pre-cultures for co-culture growth experiments were incubated in medium B with ammonium and 0.1% tryptone (Jagmann et al., 2010) for 16 h. Cells were washed in medium B without ammonium and used to inoculate co-cultures at OD_600_ = 0.001 for both strains in case of cultivation at 30°C and at OD_600_ = 0.01 for *A. hydrophila* and OD_600_ = 0.001 for *P. aeruginosa* in case of cultivation at 37°C. Co-cultures were incubated in medium B without ammonium and with suspended chitin [0.5% (w/v)] for 5 days at 30°C and for 3 days at 37°C, respectively. At 37°C and with the given inoculation values, the course of the co-culture was accelerated, but the outcome of the co-culture, i.e., pyocyanin production by *P. aeruginosa*, acetate release by, and subsequent inactivation of *A. hydrophila*, was independent of temperature. Suspended chitin was prepared as described previously (Jagmann et al., 2010).

In chitin-containing cultures bacterial growth was measured as colony forming units (CFU) grown on Pseudomonas Isolation Agar plates containing 20 μg ml^-1^ tetracycline and on LB plates as described previously (Jagmann et al., 2010). In case of soluble substrates, bacterial growth was measured as OD_600_ in a spectrophotometer (Camspec; with test-tube holder).

### Construction of Plasmids and Mutants of *P. aeruginosa*

DNA manipulations and plasmid preparations were performed according to standard methods. Genomic DNA of strain PAO1 was purified with the Puregene Tissue Core Kit B (Qiagen). Oligonucleotides used in this study are shown in Supplementary Table S2 (Supporting Information).

To construct insertional mutants of *spoT* in strains PAO1 and PAO1Δ*relA*, a fragment containing the gene was amplified from genomic DNA of strain PAO1 using the primer pair I/J. The fragment was digested with XbaI/HindIII and ligated into the corresponding sites of pEX18Ap. The resulting plasmid was linearized with PstI cutting within the ORF of *spoT* and ligated with a *res-cat-res* cassette obtained from pKO2b through digestion with PstI. The resulting plasmid was mobilized into strains PAO1 and PAO1Δ*relA* by triparental mating with *E. coli* strain DH5α as donor and strain HB101 pRK2013 as helper. Selection of mutants, excision of the vector by a second crossover, and excision of the *res-cat-res* cassette with pUCP24[*parA*] was performed as described previously (Jagmann et al., 2016).

To construct strain PAO1 deletion mutants of *ambB, relA, lysA, argH, hisD, trpB*, and a strain PAO1Δ*pchEF* deletion mutant of *pchABCD* two PCR products spanning parts of the up- and downstream regions of the respective genes were amplified from genomic DNA of strain PAO1 with primer pairs E/F and G/H for *ambB*, A/B and C/D for *relA*, T/U and V/W for *lysA*, AA/AB and AC/AD for *argH*, AE/AF and AG/AH for *hisD*, AI/AJ and AK/AL for *trpB*, and P/Q and R/S for *pchABCD*, and the resulting fragments were used as templates for a second overlapping PCR (SOE-PCR) (Ho et al., 1989) with the respective Up fw and Dn rev primer pairs. The resulting fragments were digested with XbaI/HindIII and ligated into the corresponding sites of the suicide vector pEX18Ap. Mobilization of the resulting plasmids into strains PAO1 and PAO1Δ*pchEF*, selection of mutants, and excision of the vector by a second crossover was done as described previously (Jagmann et al., 2016).

For complementation of strains PAO1Δ*spoT* and PAO1Δ*relA*Δ*spoT* with *spoT*, the gene was integrated chromosomally into the *att*Tn7 site using pUC18T-mini-Tn7T-Gm. For this, a fragment containing the putative promoter region and ORF was amplified from genomic DNA of strain PAO1 with the primer pair M/N, digested with HindIII/BamHI, and ligated into the corresponding site of pUC18T-mini-Tn7T-Gm resulting in pUC18T[*spoT*]. The plasmid was introduced in strains PAO1Δ*spoT* and PAO1Δ*relA*Δ*spoT*, respectively, by four parental mating as described previously (Jagmann et al., 2016), and integration was confirmed by PCR with the primer pair *glmS*Up/Tn7L (Choi and Schweizer, 2006). For complementation of strain PAO1Δ*relA*Δ*spoT*[*spoT*] with *relA*, the gene was integrated chromosomally into the *attB* site using mini-CTX2. For this, a fragment containing the putative promoter region and ORF was amplified from genomic DNA of strain PAO1 with the primer pair K/L, digested with HindIII/BamHI and ligated into the corresponding site of mini-CTX2. The resulting plasmid was mobilized into strain PAO1Δ*relA*Δ*spoT*[*spoT*] by triparental mating as described above, and mutants were selected on PIA plates containing tetracycline. Parts of the vector containing the tetracycline resistance cassette were removed from the genome by introducing pFLP2 as described previously (Jagmann et al., 2016), and integration was confirmed by PCR with the primer pair P_ser-up_ and P_ser-down_ (Hoang et al., 2000).

For complementation of auxotrophic strains of strain PAO1 the ORF of the respective gene was amplified with primer pairs AM/AN for *lysA*, AO/AP for *argH*, and AQ/AR for *hisD*, the resulting fragments were digested with HindIII/XbaI and ligated into the corresponding sites of pBBR1MCS5. The resulting plamids were introduced into the respective auxotrophic strain by transformation (Chuanchuen et al., 2002).

To obtain chromosomal promoter-*lacZ* reporter gene fusions of *rhlR* and *pqsA*, mini-CTX-*lacZ*[P*_rhlR_*] and mini-CTX-*lacZ*[P*_pqsA_*] were mobilized into strain PAO1Δ*relA*Δ*spoT* as described previously (Jagmann et al., 2016).

### β-Galactosidase Experiments

Expression of β-galactosidase from promoter-*lacZ* gene fusions of *rhlR* and *pqsA* was assessed in chitin-containing co-cultures of strain AH-1NΔ*lacZ* with strains PAO1 and PAO1Δ*relA*Δ*spoT*. Co-cultures were incubated as described above, and β-galactosidase activities were determined with ONPG (4 mg ml^-1^) as substrate from 800 μl sample volumes after 48 h as described previously (Jagmann et al., 2016) and calculated as *A*_405 nm_ × min^-1^ × cfu.

### Quantification of Pyocyanin

Pyocyanin in culture supernatants was quantified at 314 nm with a high-performance liquid chromatography (HPLC) system (Shimadzu) using a C_18_-reversed-phase column as described previously (Jagmann et al., 2016). Pyocyanin in supernatants of LB-grown single cultures of auxotrophic mutants and the wild-type was quantified as described previously with slight modifications (Essar et al., 1990). Briefly, 1 ml of culture supernatant was mixed with 1 ml of chloroform, and the chloroform layer was then mixed with 1 ml of 0.2 M HCl. After centrifugation, the absorption of the HCl layer was measured at 520 nm. Pyocyanin standards in LB medium were treated accordingly.

### Transposon Mutagenesis

Transposon mutagenesis of strain PAO1 with the plasmid pALMAR3 harboring a mariner transposon, screening of mutants in chitin-containing co-cultures in 96-well plates, and identification of transposon insertion sites was performed as described previously (Jagmann et al., 2016).

### Oxidative Stress Experiments

Pre-cultures of strains PAO1 and PAO1Δ*relA*Δ*spoT* were cultivated in LB medium in a volume of 3 ml in 15 ml glass tubes at 200 r.p.m. at 37°C for 16 h in a rotary shaker. Pre-cultures were used to inoculate main cultures in LB medium in a volume of 20 ml in a 100 ml Erlenmeyer flask at an OD_600_ = 0.001. Main cultures were incubated at 200 r.p.m. at 37° C for 20 h. At this timepoint, cultures had reached stationary phase and were harvested by centrifugation for 5 min at 9300 × *g*, washed with 0.9% NaCl, and resuspended in 0.9% NaCl to an OD_600_ = 1. Cell suspensions were incubated in the wells of a 24-well-plate (Sarstedt; 500 μl suspension per well) in the presence or absence of pyocyanin. Methanol served as solvent control. At distinct timepoints, CFU numbers were determined as described above.

## Results

### QS-Regulated Pyocyanin Production by *P. aeruginosa* in the Co-culture Is Not Induced by IQS-Integrated Phosphate Limitation

We could show in a previous study that pyocyanin production by *P. aeruginosa* under co-culture conditions is not caused by iron limitation. However, increasing the concentration of phosphate in the co-culture medium from 150 to 600 μM abolishes pyocyanin production by *P. aeruginosa* (Jagmann et al., 2010). It has been shown that induction of pyocyanin production in response to phosphate limitation is mediated by the newly identified QS signal molecule IQS (Lee et al., 2013), which is suggested to be a byproduct from pyochelin synthesis via PchA-F (Ye et al., 2014; Murcia et al., 2015).

**FIGURE 1:**
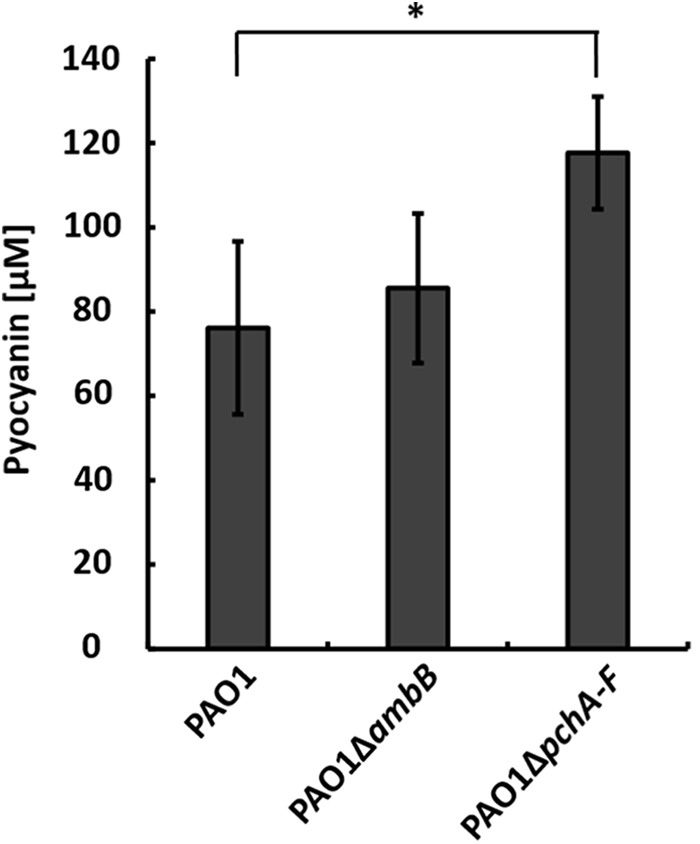
Production of pyocyanin in co-cultures with *Aeromonas hydrophila* strain AH-1N and chitin by *Pseudomonas aeruginosa* strains PAO1, PAO1Δ*ambB*, and PAO1Δ*pchA-F* after 3 days of growth. Error bars indicate standard deviation (*n* = 3 for co-cultures with strains PAO1 and PAO1Δ*ambB, n* = 5 for co-cultures with PAO1Δ*pchA-F*). Two-tailed Student’s *t*-test was performed for testing differences between groups. ^∗^Significant at *P* < 0.05.

To test whether IQS-integrated phosphate limitation caused the production of pyocyanin by strain PAO1 in the co-culture, we generated a *pchA-F* and an *ambB* mutant, which does not produce IQS as well (Lee et al., 2013), and co-cultivated them with *A. hydrophila* strain AH-1N and chitin. Both strain PAO1Δ*ambB* and PAO1Δ*pchA-F* produced pyocyanin in co-cultures. Whereas the amounts produced by strain PAO1Δ*ambB* equaled those in wild-type co-cultures, strain PAO1Δ*pchA-F* produced even higher amounts than the wild-type (**Figure 1**). These results indicated that the IQS-mediated sensing of phosphate limitation is not inducing pyocyanin production by *P. aeruginosa* in the co-culture.

### QS-Regulated Pyocyanin Production in the Co-culture Is Induced by the Stringent Response

It is likely that *P. aeruginosa* faces general nutrient limitation in the first phase of the co-culture, because the cells depend on small amounts of acetate and ammonium and other exudates that are released by *A. hydrophila* (Jagmann et al., 2010).

**FIGURE 2:**
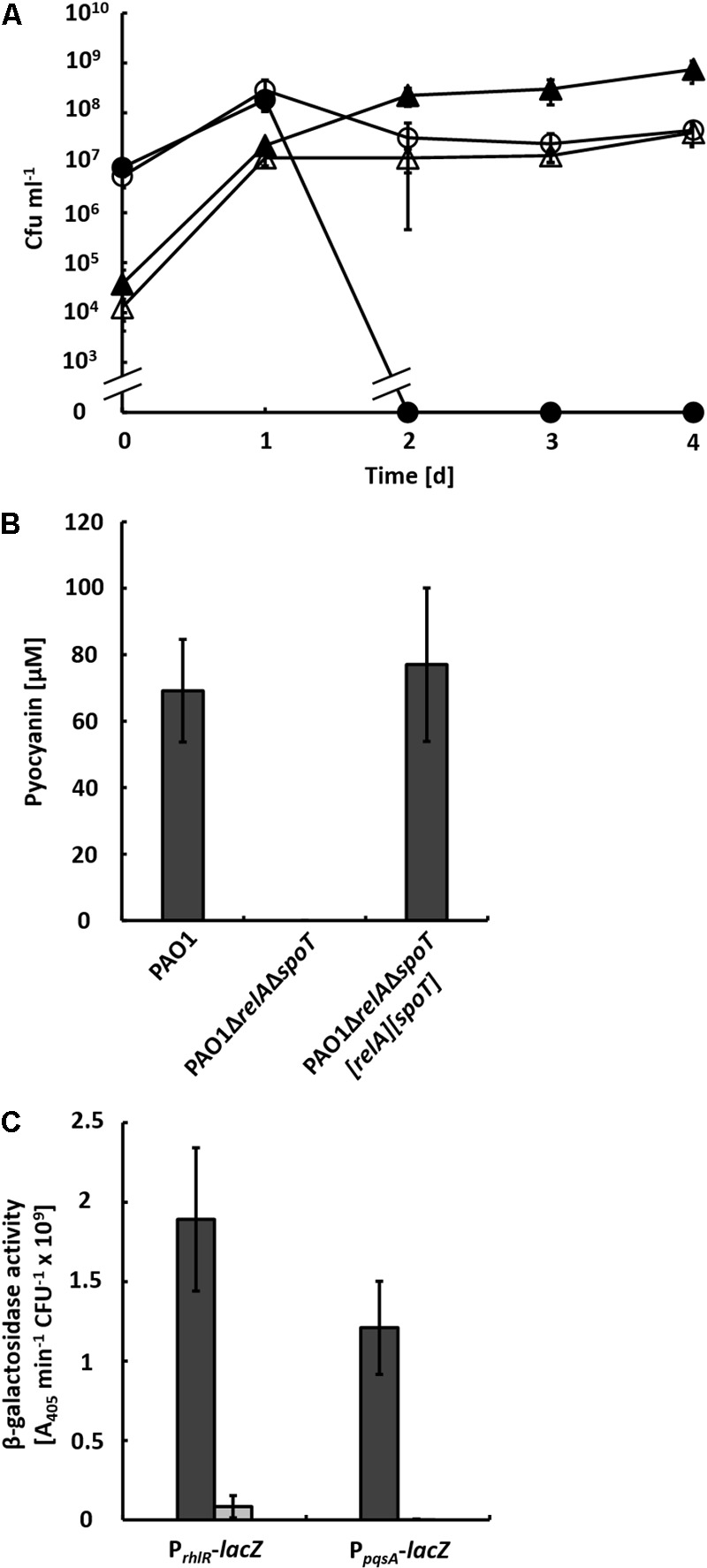
**(A)** Growth of *A. hydrophila* strain AH-1N with *P. aeruginosa* strains PAO1 and PAO1Δ*relA*Δ*spoT* in co-cultures with chitin. Colony-forming units (cfu) of strain AH-1N in co-cultures with strain PAO1 (closed circles), and with strain PAO1Δ*relA*Δ*spoT* (open circles); cfu of strain PAO1 (closed triangles); cfu of strain PAO1Δ*relA*Δ*spoT* (open triangles). **(B)** Production of pyocyanin in co-cultures with strain AH-1N and chitin by strains PAO1, PAO1Δ*relA*Δ*spoT*, and the complemented strain PAO1Δ*relA*Δ*spoT*[*relA*][*spoT*] after 3 days of growth. Error bars indicate standard deviation (*n* = 3 for co-cultures with strain PAO1, *n* = 7 for co-cultures with strains PAO1Δ*relA*Δ*spoT* and PAO1Δ*relA*Δ*spoT*[*relA*][*spoT*]). **(C)** β-Galactosidase activities of chromosomally encoded promoter-*lacZ* reporter gene fusions of *rhlR* and *pqsA* in strains PAO1 (dark gray bars) and PAO1Δ*relA*Δ*spoT* (light gray bars) in co-cultures with strain AH-1N and chitin after 2 days of growth. Error bars indicate standard deviation (*n* = 6).

To investigate, whether general nutrient limitation integrated via the stringent response is responsible for inducing QS and, in consequence, the production of pyocyanin in the co-culture, we generated the double mutant strain PAO1Δ*relA*Δ*spoT* and co-cultivated it with strain AH-1N. In these co-cultures, strain PAO1Δ*relA*Δ*spoT* reached about 10-fold lower CFU numbers compared to the wild-type and strain AH-1N was not inactivated (**Figure 2A**). No pyocyanin was produced by strain PAO1Δ*relA*Δ*spoT* (**Figure 2B**), which likely caused the survival of cells of strain AH-1N. Both pyocyanin production and inactivation of strain AH-1N by strain PAO1Δ*relA*Δ*spoT* could be restored by chromosomal integration of pUC18T[*spoT*] and mini-CTX2[*relA*], which contained the *spoT* and *relA* genes with the respective promoter regions (**Figure 2B**).

It has been shown that stringent response mutants of *E. coli* have multiple amino acid requirements and, consequently, cannot grow in minimal medium (Xiao et al., 1991). To investigate whether incubation in co-culture minimal medium somehow contributed to the lack of pyocyanin production by strain PAO1Δ*spoT*Δ*relA*, we added 0.1% tryptone as source of amino acids to the co-culture. However, strain PAO1Δ*relA*Δ*spoT* did not produce pyocyanin under these co-culture conditions as well. Additionally, unlike the *E. coli* mutants, strain PAO1Δ*relA*Δ*spoT* could grow in minimal medium with succinate (data not shown), which has been observed previously (Vogt et al., 2011). These results indicated that the lack of pyocyanin production by strain PAO1Δ*relA*Δ*spoT* was not caused by a limitation of amino acids in the co-culture medium.

It has been shown previously that during growth in LB medium in single culture expression of the PQS system is upregulated in a Δ*relA*Δ*spoT* mutant of *P. aeruginosa*, while expression of both the Las and the Rhl system is reduced (Schafhauser et al., 2014). To test whether this expression pattern could also be observed in co-culture, we introduced transcriptional promoter-*lacZ* reporter gene fusions of *pqsA* and *rhlR* into strains PAO1 and PAO1Δ*relA*Δ*spoT* and monitored β-galactosidase activities in co-cultures with these strains and strain AH-1NΔ*lacZ* (**Figure 2C**). According to β-galactosidase activities, transcription from the *rhlR* promoter was reduced in strain PAO1Δ*relA*Δ*spoT* (0.08 *A*_405 nm_ min^-1^ cfu^-1^) compared to the wild-type (1.89 *A*_405 nm_ min^-1^ cfu^-1^). However, no β-galactosidase activities of the P*_pqsA_*-*lacZ* reporter gene fusion could be measured in strain PAO1Δ*relA*Δ*spoT*. This indicated that there was no upregulation of expression of the PQS system in this mutant under co-culture conditions.

### The Stringent Response in the Co-culture Is Mediated by SpoT

**FIGURE 3:**
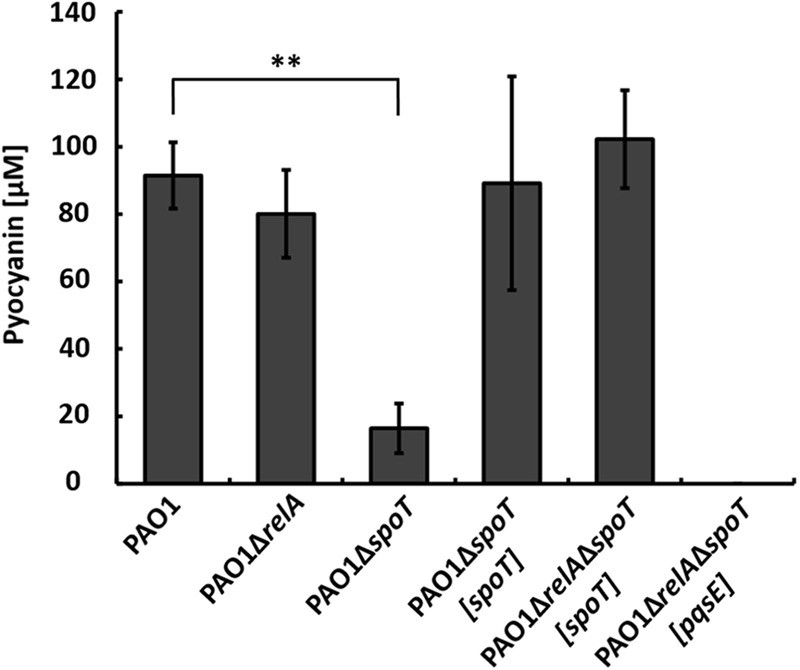
Production of pyocyanin in co-cultures with *A. hydrophila* strain AH-1N by *P. aeruginosa* strains PAO1, PAO1Δ*relA*, PAO1Δ*spoT*, the complemented strains PAO1Δ*spoT*[*spoT*], PAO1Δ*relA*Δ*spoT*[*spoT*], and strain PAO1Δ*relA*Δ*spoT*[*pqsE*] after 3 days of growth. Error bars indicate standard deviation (*n* = 3 for co-cultures with strain PAO1, *n* = 6 for co-cultures with strain PAO1Δ*relA*Δ*spoT*[*spoT*] and *n* = 8 for all other co-cultures). Two-tailed Student’s *t*-test was performed for testing differences between groups. ^∗∗^Significant at *P* < 0.0001.

To further dissect the relative contributions of RelA and SpoT, respectively, to the phenotype of strain PAO1Δ*relA*Δ*spoT* in co-culture, we constructed the respective single mutant strains PAO1Δ*relA* and PAO1Δ*spoT*. When strain PAO1Δ*relA* was co-cultivated with strain AH-1N, the same amount of pyocyanin as in wild-type co-cultures was produced indicating that stressors integrated by RelA did not contribute to the induction of pyocyanin production in the co-culture (**Figure 3**).

Despite repeated attempts, we could not obtain a Δ*spoT* single mutant when employing the gene deletion method using SOE-PCR (see “Materials and Methods” section). Therefore, we decided to generate a gene insertional mutant by inserting a *res-cat-res*-cassette conferring chloramphenicol resistance at position 1097 in the ORF of *spoT*. When mutants obtained on chloramphenicol selection plates were screened by PCR, a fragment with the correct size could only be detected in 2 of about 60 mutants. It has been discussed that such mutants may contain suppressor mutations that enable them to grow despite the lack of (p)ppGpp hydrolysis (Vogt et al., 2011; Hagmann, 2016). To investigate whether strain PAO1Δ*spoT* generated in this study contained such a mutation, we sequenced the *relA* gene of this mutant. Indeed, there was a single point mutation in the *relA* gene of strain PAO1Δ*spoT* leading to an amino acid exchange from glutamate to valine at position 359 located in the synthetase domain of RelA. When comparing the protein sequence of RelA from *P. aeruginosa* with the sequence of the catalytic fragment of the RelA/SpoT homolog Relseq of *Streptococcus dysgalactiae* subsp. *equisimilis*, for which the first crystal structure of the catalytic region was obtained (Hogg et al., 2004), this Glu359 residue was aligned to Glu336 of Relseq, which, along with other amino acids, forms H-bonds with GDP in the synthetase domain. We hypothesized that the substitution of Glu359 to a non-polar amino acid like valine would lead to a weaker binding of GDP and GTP, respectively, to the synthetase domain of RelA and, consequently, to a reduced formation of (p)ppGpp by RelA in strain PAO1Δ*spoT*, but not to a complete loss of (p)ppGpp formation as in strain PAO1Δ*relA*Δ*spoT*. To gain further insights into the role of *spoT* for induction of the stringent response in the co-culture, we tested strain PAO1Δ*spoT* in co-culture as well.

When strain PAO1Δ*spoT* was co-cultivated with strain AH-1N, pyocyanin production was reduced by about 80% compared to the wild-type, but was not completely abolished as in strain PAO1Δ*relA*Δ*spoT*. Pyocyanin production reaching wild-type levels could be restored in this mutant by complementation with chromosomally encoded *spoT*. This indicated that the stressors integrated by *spoT* were responsible for inducing QS and, thus, pyocyanin production in strain PAO1 in the co-culture. To verify this hypothesis, we complemented strain PAO1Δ*spoT*Δ*relA* with *spoT* only. When strain PAO1Δ*relA*Δ*spoT*[*spoT*] was co-cultivated with strain AH-1N, pyocyanin production was restored and reached amounts equally to those in co-cultures with the wild-type (**Figure 3**).

### Overexpression of PqsE Does Not Restore Pyocyanin Production by Strain PAO1Δ*relA*Δ*spoT* in the Co-culture

PqsE is essential for the production of pyocyanin in *P. aeruginosa*, and expression of PqsE alone is sufficient to restore pyocyanin production in a *pqsA* mutant background (Rampioni et al., 2007; Farrow et al., 2008). As we could not measure transcription from the *pqsA* promoter in strain PAO1Δ*relA*Δ*spoT* in co-cultures (**Figure 2C**), we hypothesized that no or only small amounts of PqsE were produced in this strain. To test whether production of pyocyanin by strain PAO1Δ*relA*Δ*spoT* could be restored by overexpression of PqsE, we introduced *pqsE* on plasmid pUCP18 in this strain and co-cultivated it with strain AH-1N. However, overexpression of PqsE did not restore pyocyanin production by strain PAO1Δ*relA*Δ*spoT* in co-cultures (**Figure 3**).

**FIGURE 4:**
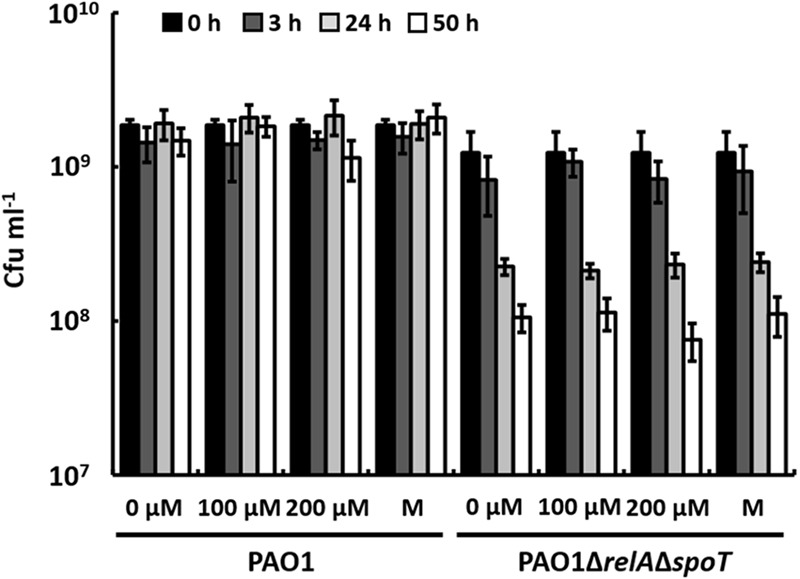
Effect of pyocyanin (0, 100, and 200 μM) and methanol (M; solvent control) on *P. aeruginosa* strains PAO1 and PAO1Δ*relA*Δ*spoT* after 0, 3, 24, and 50 h of incubation. Error bars indicate standard deviation (*n* = 3).

It has been shown previously that a Δ*relA*Δ*spoT* mutant of *P. aeruginosa* faces increased endogenous oxidative stress during biofilm cultivation, which is caused by the prooxidant effects of AQs that are overproduced as a result of the enhanced expression of the PQS system. Additionally, activities of superoxide dismutase and catalase are reduced (Nguyen et al., 2011). It has been considered unlikely, however, that phenazines like pyocyanin, which also have prooxidant functions, cause the oxidative stress in this mutant, because it does not produce phenazines. Another possible explanation for the lack of phenazine production in a Δ*relA*Δ*spoT* mutant of *P. aeruginosa* may be that the susceptibility of this mutant to oxidative stress leads to the inhibition of phenazine production by unknown regulatory mechanisms, even if PqsE is overexpressed. If this was the case, a Δ*relA*Δ*spoT* mutant should likely show sensitivity toward pyocyanin. To test this hypothesis, we prepared cell suspensions of strains PAO1 and PAO1Δ*relA*Δ*spoT* harvested in stationary phase and incubated them in the presence of different concentrations of pyocyanin (**Figure 4**). After 50 h of incubation, CFUs of the wild-type had not significantly decreased in the presence of 100 μM or 200 μM pyocyanin. In contrast, CFUs of strain PAO1Δ*relA*Δ*spoT* decreased overtime and reached a 10-fold lower number after 50 h of incubation compared to the inoculation number. This decrease of CFUs, however, occurred both in cell suspensions with and without pyocyanin indicating a general defect in long-term survival of cells of strain PAO1Δ*relA*Δ*spoT* that was not influenced by the presence of pyocyanin and the resulting putative oxidative stress within the cells.

### Genes Involved in Amino Acid Biosynthesis Are Essential for Pyocyanin Production in the Co-culture

To gain further insights into metabolic influences involved in regulation of pyocyanin production, we performed transposon mutagenesis and screened for mutants that did not produce pyocyanin in co-cultures anymore. To avoid the detection of auxotrophic mutants, we added 0.1% tryptone to the chitin-containing co-cultures, as this amount was sufficient to complement auxotrophic mutants in single cultures cultivated in minimal medium with succinate (data not shown). During these screenings, however, we again identified mutants with the transposon inserted in genes involved in amino acid biosynthesis that did not produce pyocyanin in co-culture. To study this effect further, we generated mutants with gene deletions in the amino acid biosynthesis pathways that were identified by transposon mutagenesis. We deleted genes encoding enzymes that catalyze the last step of biosynthesis of the respective amino acid, obtaining strains PAO1Δ*lysA* lacking diaminopimelate decarboxylase essential for lysine synthesis, PAO1Δ*argH* lacking argininosuccinate lyase essential for arginine synthesis, and PAO1Δ*hisD* lacking histidinol dehydrogenase essential for histidine synthesis.

**FIGURE 5:**
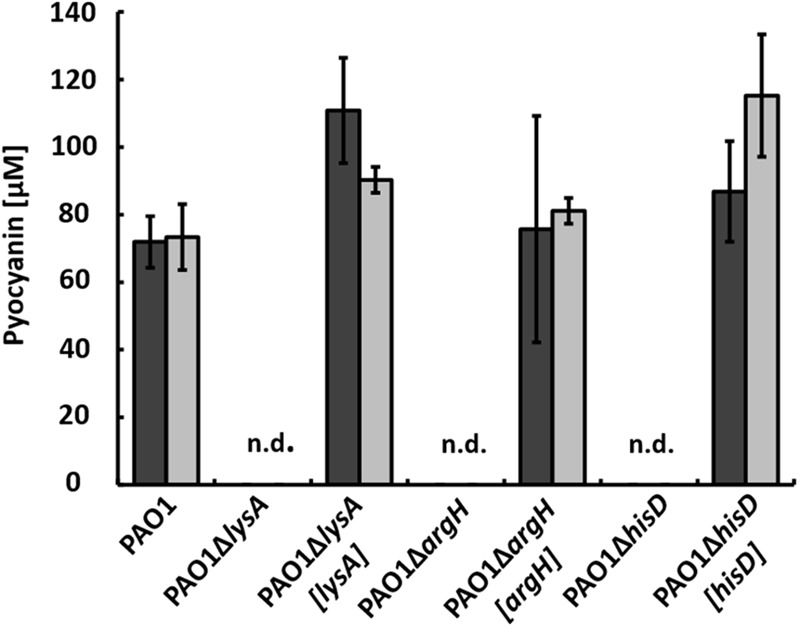
Production of pyocyanin in co-cultures with *A. hydrophila* strain AH-1N after 5 days of growth with chitin (dark gray bars) and with chitin and tryptone (light gray bars) by strain PAO1, the auxotrophic strains PAO1Δ*lysA*, PAO1Δ*argH*, PAO1Δ*hisD*, and the respective complemented strains PAO1Δ*lysA*[*lysA*], PAO1Δ*argH*[*argH*], and PAO1Δ*hisD*[*hisD*]. N.d., not detected; Error bars indicate standard deviation (*n* = 3).

When these mutants were incubated in co-cultures with strain AH-1N and chitin as sole carbon source, no pyocyanin was produced. Pyocyanin production could be restored by complementing the mutants with plasmid pBBR1MCS5 encoding the respective genes (**Figure 5**, dark gray bars).

When tryptone was added to the co-cultures, pyocyanin production by the wild-type was accelerated and cultures turned green after 24 h as described previously (Jagmann et al., 2010). Auxotrophic strains reached about the same CFU numbers compared to the wild-type in these co-cultures (data not shown), but again, no pyocyanin production could be observed despite the presence of tryptone as source of amino acids. Pyocyanin production in these co-cultures could again be restored by complementation with plasmid pBBR1MCS5 encoding the respective gene (**Figure 5**, light gray bars). To check whether auxotrophic mutants were generally unable to produce pyocyanin, we incubated strains PAO1Δ*lysA*, PAO1Δ*argH*, and PAO1Δ*hisD* in single cultures in LB medium. Under these conditions, all auxotrophic mutants produced pyocyanin in equal amounts as the wild-type (data not shown).

It might be possible that the phenotype of strains PAO1Δ*lysA*, PAO1Δ*argH*, and PAO1Δ*hisD* in co-cultures represented a general effect of amino acid auxotrophy. To support this hypothesis we generated an additional auxotrophic mutant strain PAO1Δ*trpB*, which lacks the β-chain of the tryptophan synthase. Like the other auxotrophic strains, strain PAO1Δ*trpB* did not produce pyocyanin either in co-cultures with chitin or with chitin and tryptone.

### Overexpression of PqsE Does Not Restore Pyocyanin Production by Auxotrophic Strains in Co-culture

As mentioned above, PqsE is essential for the production of pyocyanin in *P. aeruginosa* (Rampioni et al., 2007). To test whether PqsE overexpression would restore pyocyanin production by auxotrophic strains in co-cultures as well, we introduced *pqsE* on plasmid pUCP18 into strains PAO1Δ*lysA*, PAO1Δ*argH*, PAO1Δ*hisD*, and PAO1Δ*trpB*. These strains were co-cultivated with strain AH-1N in co-cultures with chitin and tryptone. However, overexpression of PqsE did not restore pyocyanin production by these strains in co-cultures.

These results indicated that amino acid auxotrophy in general was connected to the PqsE-mediated regulation of QS-controlled pyocyanin production under co-culture conditions.

## Discussion

The parasitic exploitation of the chitinolytic properties of *A. hydrophila* by *P. aeruginosa* in the co-culture is strictly dependent on the QS-controlled production of pyocyanin, which renders the co-culture a suitable model system for studying QS regulation in *P. aeruginosa*. In this study, we aimed at identifying extra- and intracellular metabolic influences that are involved in this regulation. Our results showed that an intact starvation response as well as intact amino acid biosynthesis pathways are essential for inducing QS-controlled pyocyanin production under co-culture conditions.

We focused on starvation-related regulation as the trigger for QS activation in *P. aeruginosa*, because *P. aeruginosa* likely faces nutrient limitation at the beginning of the co-culture. Such a regulatory system is the stringent response, which employs (p)ppGpp as effector molecule, and which has been shown to regulate QS in *P. aeruginosa* (van Delden et al., 2001; Schafhauser et al., 2014). Stringent response mutants of *P. aeruginosa* are impaired in pyocyanin production, when cultivated in single culture in LB medium, and are less virulent in *Drosophila melanogaster*, rat lung, and mouse infection models (Nguyen et al., 2011; Vogt et al., 2011; Xu et al., 2016). (p)ppGpp is synthetized from GTP and ATP and leads to the upregulation of stress response processes and to the downregulation of proliferative processes (Potrykus and Cashel, 2008; Kanjee et al., 2012). Like *E. coli* and other Beta- and Gammaproteobacteria, *P. aeruginosa* harbors both the (p)ppGpp synthetase RelA and the bifunctional synthetase/hydrolase SpoT (Dalebroux et al., 2010; Hauryliuk et al., 2015). We could demonstrate that SpoT mediates the activation of QS and, thus, pyocyanin production by *P. aeruginosa* in the co-culture. This was shown by two observations: first, a *relA* mutant of *P. aeruginosa* produced the same amount of pyocyanin as the wild-type in co-culture, and, second, a *relA spoT* double mutant of *P. aeruginosa* could be fully complemented by SpoT. Studies on the stringent response in both *E. coli* and *P. aeruginosa* focused mainly on RelA, whose function is well-studied. RelA is associated with the ribosomes and responds to single amino acid limitation by sensing uncharged tRNAs (Hauryliuk et al., 2015; Arenz et al., 2016; Brown et al., 2016). SpoT, in contrast, responds to a variety of starvation stresses in *E. coli*, e.g., carbon, iron, and phosphate starvation, but the molecular details of SpoT regulation are still unknown (Hauryliuk et al., 2015). In *P. aeruginosa*, the stringent response mediated by SpoT has been shown to be essential for the detection of fatty acid starvation via activation of SpoT by the acyl carrier protein AcpP, for the expression of *usp* genes encoding universal stress proteins, and for full virulence in eukaryotic infection models (Boes et al., 2008; Battesti and Bouveret, 2009; Vogt et al., 2011). In these studies, a single environmental trigger has not been demonstrated, and besides binding to AcpP during fatty acid starvation the exact mechanisms by which these triggers are integrated into SpoT activation are unknown.

In our co-culture model system, the exact environmental stress signal that activates the SpoT-mediated stringent response is not known as well. However, it is very likely that the stringent response is activated in response to nutrient starvation. In the co-culture, *P. aeruginosa* faces both carbon and nitrogen starvation due to the lack of an utilizable substrate and of ammonium, and it is dependent on acetate and ammonium that are transiently released by *A. hydrophila*. We have shown that increasing the amount of phosphate in the co-culture abolishes pyocyanin production by *P. aeruginosa* (Jagmann et al., 2010), suggesting that the amounts of acetate and ammonium released by *A. hydrophila* are sufficient to suppress starvation signaling, and that *P. aeruginosa* rather faces phosphate starvation in the co-culture under normal cultivation conditions. In *P. aeruginosa*, phosphate limitation is integrated via the two component system PhoR-PhoB (Jensen et al., 2006). It has been demonstrated in *E. coli*, that phosphate starvation results in SpoT-dependent accumulation of (p)ppGpp, and that this accumulation is diminished in a *phoB* mutant showing a connection of phosphate sensing and the stringent response (Spira and Yagil, 1998). If phosphate limitation was the trigger for QS activation in *P. aeruginosa* in the co-culture, our results indicate, however, that IQS signaling is not involved in this regulation as an IQS negative mutant produced the same amount of pyocyanin compared to the wild-type. IQS has been shown to connect phosphate stress-response mechanisms to the Rhl and PQS systems (Lee et al., 2013). In this work, however, the stringent response was not addressed, and it may be possible that phosphate stress is integrated differently into QS under differential culture conditions. We cannot exclude, however, that activation of QS in and the production of pyocyanin by *P. aeruginosa* in the co-culture depended on the combination of several starvation signals that concertedly induced the SpoT-mediated stringent response.

In *E. coli*, SpoT exhibits a weak synthetic and strong hydrolytic activity for (p)ppGpp (An et al., 1979; Xiao et al., 1991). We assume that the balance of these activities was shifted to (p)ppGpp synthesis upon starvation in the co-culture, so that (p)ppGpp accumulation is independent of RelA. It has been hypothesized that the hydrolytic activity of SpoT is important for balancing the intracellular concentrations of (p)ppGpp produced by RelA during growth (An et al., 1979; Xiao et al., 1991; Hauryliuk et al., 2015). Therefore, a disruption of *spoT* is lethal in *E. coli*. The *spoT* gene has been designated as essential gene in *P. aeruginosa* as well (Hashimoto et al., 2005), and this is supported by the fact that we obtained *spoT* mutants of *P. aeruginosa* only, when there was a point mutation in *relA*. Failed attempts to generate a Δ*spoT* mutant of *P. aeruginosa* and other bacteria have been described earlier (Fisher et al., 2005; Manuel et al., 2011, 2012; Vogt et al., 2011; Holley et al., 2014; Hagmann, 2016). However, there are also studies that describe the generation of *spoT* mutants of *P. aeruginosa* (Viducic et al., 2006; Xu et al., 2016) and of other Pseudomonads (Takeuchi et al., 2012; Chatnaparat et al., 2015). To our knowledge, possible suppressor mutations, for example in *relA*, were not investigated in these studies.

We could show previously that both the Rhl and PQS system are crucial for pyocyanin production by *P. aeruginosa* in the co-culture (Jagmann et al., 2016). During cultivation of a *relAspoT* mutant of *P. aeruginosa* in LB medium in single culture, expression of the PQS system is upregulated, whereas the Rhl system is not fully expressed (Schafhauser et al., 2014). As shown by transcriptional *lacZ* fusion studies, expression of RhlR was downregulated in strain PAO1Δ*relA*Δ*spoT* in the co-culture as well. However, no upregulation of *pqsA* expression could be observed. Transcription of the *pqsABCDE* operon is negatively and positively regulated by RhlR and by PqsR, respectively, which bind to two distinctive start sites (Dötsch et al., 2012; Brouwer et al., 2014). Increased expression of the *pqsABCDE* operon would depend on transcription from the PqsR transcriptional start site. In our study we monitored expression from this PqsR-controlled promoter, indicating that the observed absence of β-galactosidase activity indeed originated from the absence of PqsR-induced *pqsABCDE* expression. It has been suggested that a third transcript comprising *pqsD, pqsE*, and the adjacent operon *phnAB* occurs during growth of *P. aeruginosa* in minimal medium, which would elevate the expression of PqsE and, in consequence, of PqsE-controlled virulence factors such as pyocyanin (Knoten et al., 2014). We cannot exclude that such a transcript occurs during growth under co-culture conditions as well. However, the *relAspoT* mutant produced no pyocyanin in the co-culture suggesting the absence of PqsE. In addition, it is known that QS induction in *P. aeruginosa* varies depending on environmental conditions, and the composition of the VGA minimal medium used in the study by Knoten et al. (2014) differs from the minimal medium B used in our experiments. Thus, it is feasible that under co-culture conditions, expression of both the PQS and the Rhl system is downregulated resulting in a lack of pyocyanin production.

While searching for intracellular metabolic influences on QS regulation, we identified amino acid auxotrophic strains of *P. aeruginosa* that did not produce pyocyanin in co-cultures even in the presence of tryptone as amino acid source. It has been hypothesized that intracellular metabolic fluxes play a role in starvation sensing in *P. aeruginosa* (Mellbye and Schuster, 2014), and amino acid biosynthesis pathways might be involved in such regulation.

In a *pqsA* mutant of *P. aeruginosa*, overexpression of PqsE restores pyocyanin production (Farrow et al., 2008). In contrast, overexpression of PqsE could not restore pyocyanin production by both strain PAO1 Δ*relA*Δ*spoT* and the auxotrophic mutants in co-cultures. Thus, we have identified two physiological conditions, under which *P. aeruginosa* is not responsive to PqsE overexpression. It has been observed before that a *relAspoTpqsA* triple mutant does not produce pyocyanin in the presence of PqsE (Nguyen et al., 2011). Concerning the auxotrophic mutants, it has been observed that in a *pheA* mutant of *P. aeruginosa* strain PA14 pyocyanin production could not be restored by PqsE overexpression as well (Brouwer, 2015). The chorismate mutase PheA catalyzes both the conversion of chorismate to prephenate and of prephenate to phenylpyruvate in the pathway of phenylalanine biosynthesis. The auxotrophic mutants investigated in our study were defect in the biosynthesis of amino acids that branch off different intermediates in the central metabolism. This suggests that the inability to produce pyocyanin even though PqsE is overexpressed might be a general effect of the inability to synthesize amino acids.

Constitutive expression of *pqsE* from plasmid pUCP18 is under the control of the *lac* promoter. Therefore, we hypothesize that a *pqsE* mRNA is at one point present in the cell, and that inhibition of PqsE-activated pyocyanin production is caused either by posttranscriptional regulation of PqsE or by regulation of pyocyanin biosynthesis genes. It has been suggested that the regulatory activity exerted by PqsE depends on the presence of the protein and is not caused by a *pqsE* regulatory RNA being involved in the expression of PqsE-controlled genes. However, regulatory mechanisms controlling gene expression that require the PqsE protein remain to be elucidated (Rampioni et al., 2016). It has been shown that PqsE represses the transcription of the *pqsABCDE* operon, and this effect is enhanced when PqsE is overexpressed (Rampioni et al., 2010). However, this negative autoregulatory role of PqsE should not be involved in the pyocyanin negative phenotype of the *relAspoT* mutant and the auxotrophic mutants overexpressing PqsE in the co-culture, because pyocyanin production caused by PqsE overexpression is independent of quinolone synthesis (Farrow et al., 2008), and because expression of *pqsE* from plasmid pUCP18 is independent of its native promoter.

Pyocyanin is synthesized via the products of the biosynthetic operons *phzA1-G1* and *phzA2-G2* that are expressed from differentially regulated promoter regions. It has been shown that the *phz* genes are upregulated when PqsE is overexpressed in the *P. aeruginosa* wild-type (Rampioni et al., 2010). However, knowledge about the connection between PqsE and metabolic influences, respectively, and the regulation of these operons is still scarce.

Taken together, by employing the co-culture model system, we have identified metabolic influences that are involved in QS regulation in *P. aeruginosa*. Both the stringent response and biosynthesis of amino acids have to be functional for QS-controlled pyocyanin production under these conditions. The previously discovered function of GbuA for the induction of pyocyanin biosynthesis in the co-culture might be linked to nutrient starvation and amino acid metabolism as well as this enzyme has been shown to be involved in an alternative arginine degradation pathway, which might be induced under starvation for the breakdown of endogenous proteins (Jagmann et al., 2016). As the co-culture represents conditions that do also prevail in human infections, that is nutrient limitation and competition with other bacteria (Harrison, 2007; Hogardt and Heesemann, 2010), regulatory mechanisms that play a role in the co-culture might also be important in infection processes.

## Author Contributions

NJ conceived and performed the experiments, analyzed and interpreted the data, and wrote the manuscript. BP interpreted the data and commented on the manuscript.

## Conflict of Interest Statement

The authors declare that the research was conducted in the absence of any commercial or financial relationships that could be construed as a potential conflict of interest.
