# Supplementary material for: SpoT-Mediated Regulation and Amino Acid Prototrophy Are Essential for Pyocyanin Production During Parasitic Growth of Pseudomonas aeruginosa in a Co-culture Model System With Aeromonas hydrophila
Source: Front Microbiol. 2018 Apr 18;9:761. doi: 10.3389/fmicb.2018.00761 (PMC5915560; doi:10.3389/fmicb.2018.00761)
Supplement: Supplementary file 1 [file Table_1.docx]

**Supplementary Table S1.** Strains and plasmids used in this study**.**

| Strains and plasmids | Relevant characteristics | Source or reference |
| --- | --- | --- |

*Pseudomonas aeruginosa*

PAO1 PAO1 Nottingham wildtype Holloway collection

PAO1∆*relA* PAO1 with 1908 bp deletion of *relA* This study

PAO1∆*spoT* PAO1 with *res*-sites inserted into This study

unique PstI site of *spoT*

PAO1∆*relA*∆*spoT* PAO1 with complete deletion of This study

*relA* and *res*-sites inserted into

unique PstI site of *spoT*

PAO1∆*ambB* PAO1 with 1698 bp deletion of This study

*ambB*

PAO1∆*pchEF pchEF* deletion mutant, pyochelin Ghysels *et al.*, 2004

negative

PAO1∆*pchABCDEF* PAO1∆*pchEF* with complete This study

Deletion of *pchABCD*

PAO1∆*lysA* PAO1 with complete deletion of *lysA* This study PAO1∆*argH* PAO1 with complete deletion of *argH* This study

PAO1∆*hisD* PAO1 with complete deletion of *hisD* This study

PAO1∆*trpB* PAO1 with complete deletion of *trpB* This study

PAO1∆*relA*[miniTn7] PAO1∆*relA* with unmarked mini-Tn7 This study

insertion at *att*Tn7 site

PAO1∆*relA*[*relA*] PAO1∆*relA* with *relA* This study

complementation inserted at *att*Tn7 site

PAO1∆*spoT*[miniTn7] PAO1∆*spoT* with unmarked mini-Tn7 This study

insertion at *att*Tn7 site

PAO1∆*spoT*[*spoT*] PAO1∆*spoT* with *spoT* This study

complementation inserted at *att*Tn7 site

PAO1∆*relA*∆*spoT* PAO1∆*relA*∆*spoT* with unmarked This study

[miniTn7] mini-Tn7 insertion at *att*Tn7 site

PAO1∆*relA*∆*spoT*[*spoT*] PAO1∆*relA*∆*spoT* with *spoT* This study

complementation at *att*Tn7 site

PAO1∆*relA*∆*spoT*[*relA*] PAO1∆*relA*∆*spoT* with *relA* This study

complementation at *att*Tn7 site

PAO1∆*relA*∆*spoT* PAO1∆*relA*∆*spoT* with unmarked This study

[miniTn7][miniCTX2] mini-Tn7 insertion at *att*Tn7 site and

miniCTX2 insertion at *attB* site

PAO1∆*relA*∆*spoT* PAO1∆*relA*∆*spoT* with *spoT* This study

[*relA*][*spoT*] complementation at *att*Tn7 site and

*relA* complementation at *attB* site

PAO1[*lacZ*] PAO1 *attB*::(mini-CTX-*lacZ*) Jagmann *et al*., 2016 PAO1[P*_rhlR_*-*lacZ*] PAO1 *attB*::(P*_rhlR_*-*lacZ*) Jagmann *et a*l., 2016

PAO1[P*_pqsA_*-*lacZ*] PAO1 *attB*::(P*_pqsA_*-*lacZ*) Jagmann *et al*., 2016

PAO1∆*relA*∆*spoT*[*lacZ*] PAO1∆*relA*∆*spoT* This study

*attB*::(mini-CTX-*lacZ*)

PAO1∆*relA*∆*spoT* PAO1∆*relA*∆*spoT* *attB*::(P*_rhlR_*-*lacZ*) This study

[P*_rhlR_*-*lacZ*]

PAO1∆*relA*∆*spoT* PAO1∆*relA*∆*spoT* *attB*::(P*_pqsA_*-*lacZ*) This study

[P*_pqsA_*-*lacZ*]

*Aeromonas hydrophila*

AH-1N AH-1N wildtype Swift *et al.*, 1999

AH-1N∆*lacZ* AH-1N with complete deletion of Jagmann *et al*., 2016

*lacZ*

*Escherichia coli*

DH5a *recA1 endA1 hsdR17 thi-1* Sambrook and

*supE44 gyrA96 relA1 deoR* Russell, 2001

*Δ(lacZYA-argF) U196*

(Φ80*lacZ*ΔM15)

HB101 *thi*-1 *hsd* S20 (rB–, mB–) *sup*E44 Promega

*rec*A13 *ara*-14 *leu*B6 *pro*A2

*lac*Y1*rpsL*20 *(*strr) *xyl*-5 *mtl*-1

*gal*K2

Plasmids

pEX18Ap Gene replacement vector, Ap^R^, Hoang *et al.*, 1998

*sacB*

pEX18Ap[*relA*] pEX18Ap with *relA* deletion This study

cassette as XbaI-HindIII fragment

pEX18Ap[*spoT*::*Cm*] pEX18Ap carrying *spoT* with This study

*res-cat-res* cassette from pKO2b

inserted into unique PstI site of

*spoT*

pEX18Ap[lysA] pEX18Ap with *lysA* deletion This study

cassette as XbaI-HindIII fragment

pEX18Ap[argH] pEX18Ap with *argH* deletion This study

cassette as XbaI-HindIII fragment

pEX18Ap[hisD] pEX18Ap with *hisD* deletion This study

cassette as XbaI-HindIII fragment

pEX18Ap[trpB] pEX18Ap with *trpB* deletion This study

cassette as XbaI-HindIII fragment

pKO2b pUC18Sfi containing a *res-cat-res* Klebensberger *et al.*,

cassette, Ap^r^, Cm^R^ 2009

pRK2013 Helper plasmid for triparental Figurski and

conjugation; IncP Tra^+^ Km^R^ Helinski, 1979

pFLP2 Source of Flp recombinase, Ap^R^ Becher and

Schweizer, 2000

pUCP24[*parA*] *parA* as EcoRI-HindIII fragment in Smits *et al.*, 2002

pUCP24, Gm^R^

pUX-BF13 helper plasmid providing the Tn7 Bao et al., 1991

transposition function in trans, Ap^R^,

mob^+^

mini-CTX-*lacZ* cloning vector for transcriptional *lacZ* Becher and

fusions, Tc^R^ Schweizer, 2000

mini-CTX-*lacZ*[P*_rhlR_*] mini-CTX-*lacZ* with a 162 bp Jagmann *et al*., 2016

fragment of the *rhlR* promoter

mini-CTX-*lacZ*[P*_pqsA_*] mini-CTX-*lacZ* with 199 bp-fragment Jagmann *et al*., 2016

of *pqsA* promoter region between

BamHI and SalI

pUC18T-mini-Tn7T-Gm suicide delivery plasmid for Choi and

*P. aeruginosa* containing Schweizer, 2006

mini-Tn7 elements, Gm^R^

pUC18T[*relA*] pUC18T-mini-Tn7T-Gm with HindIII- This study

BamHI fragment containing ORF

and promoter region of *relA*

pUC18T[spoT] pUC18T-mini-Tn7T-Gm with HindIII- This study

BamHI fragment containing ORF

and promoter region of *spoT*

mini-CTX2 integration vector, Tc^R^ Hoang *et al.*, 2000

mini-CTX2[*relA*] mini-CTX2 with BamHI-HindIII This study

fragment containing ORF and

promoter region of *relA*

pBBR1MCS5 Broad-host-range cloning vector, Gm^R^ Kovach *et al.*, 1995

pBBR1[lysA] pBBR1MCS5 with HindIII-XbaI This study

fragment containing the ORF of *lysA*

pBBR1[argH] pBBR1MCS5 with HindIII-XbaI This study

fragment containing the ORF of *argH*

pBBR1[hisD] pBBR1MCS5 with HindIII-XbaI This study

fragment containing the ORF of *hisD*

pUCP18::*pqsE Escherichia–Pseudomonas* shuttle Rampioni *et al.*, 2010

vector for *pqsE* complementation;

Ap^R^

Bao, Y., Lies, D. P., Fu, H., and Roberts, G. P. (1991). An improved Tn7-based system for the single-copy insertion of cloned genes into chromosomes of gram-negative bacteria. *Gene* 109, 167-168.

Becher, A., and Schweizer, H.P. (2000). Integration-proficient *Pseudomonas aeruginosa* vectors for isolation of single-copy chromosomal *lacZ* and *lux* gene fusions. *Biotechniques* 29**,** 948-950.

Figurski, D.H., and Helinski, D.R. (1979). Replication of an origin-containing derivative of plasmid RK2 dependent on a plasmid function provided in trans. *Proc Natl Acad Sci USA* 76**,** 1648-1652.

Ghysels, B., Dieu, B.T.M., Beatson, S.A., Pirnay, J.P., Ochsner, U.A., Vasil, M.L., *et al.* (2004). FpvB, an alternative type I ferripyoverdine receptor of *Pseudomonas* *aeruginosa*. *Microbiology* 150**,** 1671-1680. doi: [10.1099/mic.0.27035-0](https://doi.org/10.1099/mic.0.27035-0)

Hoang, T.T., Karkhoff-Schweizer, R.R., Kutchma, A.J., and Schweizer, H.P. (1998). A broad-host-range Flp-FRT recombination system for site-specific excision of chromosomally-located DNA sequences: application for isolation of unmarked *Pseudomonas aeruginosa* mutants. *Gene* 212**,** 77-86.

Klebensberger, J., Birkenmaier, A., Geffers, R., Kjelleberg, S., and Philipp, B. (2009). SiaA and SiaD are essential for inducing autoaggregation as a specific response to detergent stress in *Pseudomonas aeruginosa*. *Environ Microbiol* 11**,** 3073-3086. doi: [10.1111/j.1462-2920.2009.02012.x](https://doi.org/10.1111/j.1462-2920.2009.02012.x)

Kovach, M.E., Elzer, P.H., Hill, D.S., Robertson, G.T., Farris, M.A., Roop, R.M., *et al*. (1995). 4 new derivatives of the broad-host-range cloning vector pBBR1MCS, carrying different antibiotic-resistance cassettes. *Gene* 166**,** 175-176.

Sambrook, J., and Russell, D.W. (2001). *Molecular Cloning: A laboratory manual.* New York: Cold Spring Harbor Laboratory Press.

Smits, T.H.M., Balada, S.B., Witholt, B., and Van Beilen, J.B. (2002). Functional analysis of alkane hydroxylases from gram-negative and gram-positive bacteria. *J Bacteriol* 184**,** 1733-1742.

Swift, S., Lynch, M.J., Fish, L., Kirke, D.F., Tomas, J.M., Stewart, G.S.a.B., *et al*. (1999). Quorum sensing-dependent regulation and blockade of exoprotease production in *Aeromonas hydrophila*. *Infect Immun* 67**,** 5192-5199.
