# Supplementary material for: SpoT-Mediated Regulation and Amino Acid Prototrophy Are Essential for Pyocyanin Production During Parasitic Growth of Pseudomonas aeruginosa in a Co-culture Model System With Aeromonas hydrophila
Source: Front Microbiol. 2018 Apr 18;9:761. doi: 10.3389/fmicb.2018.00761 (PMC5915560; doi:10.3389/fmicb.2018.00761)
Supplement: Supplementary file 2 [file Table_2.docx]

**Supplementary Table S2.** Oligonucleotides used in this study. Restriction sites are underlined.

| Oligonucleotides | Sequence 5’->3’ |  |
| --- | --- | --- |

Primer

A relA Up fw AAATCTAGAAGCTCTGGCTGCAAGGTGCG

B relA Up rev CACCACCGGCCCGAAATGCT

C relA Dn fw AGCATTTCGGGCCGGTGGTGGGCGAGGCGGAAACAGGC

D relA Dn rev ATTAAGCTTCCAGCCCCTCGCGAACCTTG

E ambB Up fw ATTTCTAGAGAGATCACCCTGGGCTTTCC

F ambB Up rev GACCAGCACCAGCAGGCAG

G ambB Dn fw CTGCCTGCTGGTGCTGGTCGAGCGGTTGCTGGAGGATG

H ambB Dn rev TTAAAGCTTTCCTGGTGTTCGAGCAGTTC

I spoT fw AAAATCTAGAGGGTGAACCCTTGCCGG

J spoT rev TTTAAGCTTACACAGGAAAAAGCGGGTCA

K relA komp fw TTTTTTTAAGCTTGAGGCGGTACGCGAAATGAG

L relA komp rev TTTTTTTGGATCCAGGTCGTCGAGTCGGTACAT

M spoT komp fw TTTTTTTAAGCTTCCGTTGAAGACTGCCTGGACAA

N spoT komp rev TTTTTTTGGATCCGGCGTTGTCGGTGTGGATAACGG

P pchABCD Up fw aaatctagaCCGAGCTGGACGATGGTGTA

Q pchABCD Up rev GCGATCTCCGTGGATGCGG

R pchABCD Dn fw CCGCATCCACGGAGATCGCAAACGAAGACCCCCTGCG

S pchABCD Dn rev tttAAGCTTGCGGATGCTGAACCTTAGGA

T lysA Up fw aaatctagaTCGAAGGCGTCTTCCACCTG

U lysA Up rev GGGCGCTCTCTCAGAAACCGT

V lysA Dn fw acggtttctgagagagcgcccGGCCCAGACCATGCTTT

TGC

W lysA Dn rev tttAAGCTTCGCTGGCCATTCACCTCTAT

AA argH Up fw aaatctagaCCACTGGCTCTGCAGGTAAA

AB argH Up rev GGTATCTCTCGCTGCAACAA

AC argH Dn fw ttgttgcagcgagagataccAGTCGCGCCCAGTGCCC

AD argH Dn rev tttaagcttGCTACGGCAGCCACGACTAT

AE hisD Up fw aaatctagaCATCGTACCGTCGGAGAACC

AF hisD Up rev AGCGAGCCTCTTTTATGGATG

AG hisD Dn fw catccataaaagaggctcgctGCATGAGCAAATTCTG

GAG

AH hisD Dn rev tttaagcttGACTACCACCACCGAATCC

AI trpB Up fw aaatctagaCGCTTCCAGCAGGTAGTAGAG

AJ trpB Up rev GGAGGGCTCCAGGAAATGAC

AK trpB Dn fw gtcatttcctggagccctccGCCGCCTGCAGACCCGC

AL trpB Dn rev tttaagcttTTCCTCGACGTGTTCCAGGG

AM lysA fw tttaagcttGTTTCTGAGAGAGCGCCCAT

AN lysA rev aaatctagaTCTTGGTGAAGCGCAAAAGC

AO argH fw tttaagcttGAGAGATACCATGAGCGTAGAG

AP argH rev aaatctagaGCGATCAAGTCTCGGGTAGG

AQ hisD fw tttaagcttGAGGCTCGCTATGACCGC

AR hisD rev aaatctagaTCCTTGACGAAGGGACTCCA
